# Thymic B Cells and Central T Cell Tolerance

**DOI:** 10.3389/fimmu.2015.00376

**Published:** 2015-07-22

**Authors:** Tomoyoshi Yamano, Madlen Steinert, Ludger Klein

**Affiliations:** ^1^Institute for Immunology, Ludwig-Maximilians-University Munich, Munich, Germany

**Keywords:** central tolerance, B cells, antigen presentation, germinal center, CD40, class-switching, Aire

## Abstract

Central T cell tolerance is believed to be mainly induced by thymic dendritic cells and medullary thymic epithelial cells. The thymus also harbors substantial numbers of B cells. These may arise though intrathymic B lymphopoiesis or immigration from the bloodstream. Importantly, and in contrast to resting “mainstream” B cells in the periphery, thymic B cells display elevated levels of MHC class II and constitutively express CD80. Arguably, their most unexpected feature is the expression of autoimmune regulator. These unique features of thymic B cells result from a licensing process that involves cross-talk with CD4 single-positive T cells and CD40 signaling. Together, these recent findings suggest that B cells play a more prominent role as thymic APCs than previously appreciated.

## Introduction

B cells represent approximately 0.3% of the thymic cellularity. Although their absolute number may, in fact, exceed that of thymic dendritic cells, their role a APCs for central tolerance induction is not well understood, and thymic B cells have often been regarded as “innocent bystanders.” Recent data suggest that this view may need to be revised. Here, we will provide a short overview of novel insights into distinct features of thymic B cells and how these may predispose thymic B cells to support T cell tolerance.

## The Origin of Thymic B Cells

### Intrathymic B cell development

The early thymic progenitor (ETP), i.e., the cell type that gives rise to the T cell lineage, retains some B cell potential (1). Notch signaling is essential for T lineage specification in ETPs, so that precursors lacking Notch-1 fail to generate T lineage cells in the thymus. There are increased numbers of B cells in the thymus of conditional Notch-1 knockout mice (2), and this was interpreted to indicate that in the absence of Notch signaling, ETPs undergo B cell differentiation as a “default cell fate” (3, 4). However, subsequent experiments argued that most of the accumulation of thymic B cells under these conditions is not a cell intrinsic effect of Notch-deficiency, but may stem from immigration of peripheral B cells as a result of excessive niche availability (5). This notion is supported by the observation that perturbations in T cell differentiation downstream of the loss of B lineage potential result in effects on thymic B cell numbers that are reciprocal to their effects on T cell numbers. Specifically, TCRβ^−/−^ mice, in which T cell development is blocked prior to pre-TCR expression, harbor a drastically diminished overall T cell compartment (arrested at the DN3 stage), yet have a 10-fold increase in thymic B cell numbers, whereas TCRα^−/−^ mice, in which T cell differentiation is arrested at the DP stage, display slightly decreased numbers of B cells in the thymus (6, 7).

Does intrathymic B cell differentiation occur under non-perturbed *steady state* conditions? There were early reports on the existence of cells within the thymus whose phenotype – surface (s)IgM^−^B220^+^CD43^+^ – resembled that of B cell progenitors in the bone marrow (BM). When these cells were purified and injected intrathymically (i.t.), they gave rise to mature B cells within the thymus (8). Akashi et al. estimated that concomitant to the release of about 1 × 10^6^ T cells, the thymus also exports around 3 × 10^4^ B cells each day (6). In sum, there is good evidence that part of the thymic B cell population arises through differentiation within the thymus.

### Immigration of peripheral B cells

Using more conclusive surface marker combinations, we recently revisited the issue whether the thymus harbors significant numbers of B cell precursors (9). Among CD19^+^IgM^−^IgD^−^BM cells, pre- and pro-B cells are commonly identified as CD2^+^c-Kit^−^ and CD2^−^c-Kit^+^ cells, respectively. We found that around one-third of thymic CD19^+^ cells were surface IgM^−^IgD^−^, and thereby resembled B cell precursors in the BM. However, pro-B cells (CD19^+^IgM^−^IgD^−^CD2^−^c-Kit^+^) were essentially undetectable in the thymus. Moreover, most thymic CD19^+^IgM^−^IgD^−^CD2^+^c-Kit^−^ cells expressed surface sIgG. Thus, the majority of CD19^+^IgM^−^IgD^−^cells in the thymus (unlike their phenotypic counterparts in the BM) are class-switched mature B cells and not B cell precursors. Based upon the paucity of B cell precursors in the thymus, we wondered whether peripheral B cells enter the thymus in the *steady state*. In order to address this, we intravenously injected bulk splenic B cells into syngeneic hosts. Seven days later, donor B cells were detectable in both spleen and thymus, whereby the relative abundance among host B cells in the thymus was about 5- to 10-fold lower as compared to the spleen. Although at first glance, this suggests that thymic immigration is a fairly efficient process, any comparison of its efficacy in relation to homing to the spleen is blurred by the fact that upon entering the thymus, B cells undergo several cell divisions. The capacity to enter the thymus does not seem to be restricted to any particular activation state, since purified naïve B cells (IgM^+^IgD^+^) also entered the thymus.

Taken together, it is reasonable to assume that both intrathymic B lymphopoesis and immigration of BM-derived B cells contribute to the thymic B cell pool. However, we lack a precise understanding of the relative contribution of either pathway. On the one hand, the virtual absence of pro- and pre-B cells may render intrathymic differentiation an unlikely source of the majority of thymic B cells. On the other hand, thymic B cells in parabiosed mice do not equilibrate to the same extent as is observed for splenic B cells, insinuating a substantial contribution of intrathymic B cell differentiation (10). Unraveling the lineage relation between peripheral “mainstream” B cells and thymic B cells remains experimentally challenging. Ultimately, this issue is linked to the questions whether thymic B cells display distinct features, and whether these features are manifestations of a hard-wired “thymic B lineage differentiation program” or result from extrinsic cues.

## Intrathymic B Cell Licensing

### The unusual phenotype of thymic B cells

Some phenotypic features (e.g., CD5 expression) had suggested that thymic B cells may be related to the fetal liver-derived B1 lineage (8). However, whereas *bona fide* B1 cells in the peritoneal cavity are restored only by reconstitution with fetal liver cells, but not BM cells, the thymic B cell pool is efficiently generated from both precursors (10). Thus, thymic B cells clearly are genealogically related to the B2 “mainstream” B cell lineage.

Unlike resting B cells in spleen and lymph node, thymic B cells express high levels of MHC class II and the co-stimulatory molecules CD80 and CD86 (9–11). Moreover, a substantial fraction of thymic B cells have class-switched, whereby the distribution of isotype classes is remarkably stereotypic from mouse to mouse. Perhaps the most unusual feature of thymic B cells is their expression of the autoimmune regulator (Aire) gene. Aire is known to be crucial for “promiscuous gene expression” (pGE) of peripheral self-antigens in medullary thymic epithelial cells (mTECs) (12). The only cell-type other than mTECs that had so far been reported to express Aire is rare cells in the lymph node which have been termed as extrathymic Aire expressing cells (eTACs) (13). eTACs are of hematopoietic origin, yet their exact lineage identity remains elusive (14). Using Aire-reporter mice, we noted a reporter-positive population of non-mTEC cells in the thymus and subsequently identified these cells as thymic B cells (9). Faithful expression of the Aire-reporter was confirmed by RT-PCR and intracellular protein staining. Aire protein was detectable in nuclear dots in around 2–3% of thymic B cells, whereby protein levels were substantially lower than in mTECs. A comparison of gene expression profiles in WT versus Aire^−/−^ thymic B cells revealed that several hundred genes are differentially expressed. Very few of these had previously been reported to be Aire dependent in mTECs or eTACs, indicating that Aire’s function as a transcriptional regulator is cell context dependent. Of note, whereas in mTECs the expression of several thousand genes is modulated by Aire, only a few hundred genes are controlled by Aire in thymic B cells or eTACs. Furthermore, it remains to be established whether Aire-dependent expression of any tissue-restricted antigen in thymic B cells is essential for T cell tolerance.

Are these distinctive features of thymic B cells an inherent feature of B cells that arise through intrathymic B lympopoiesis? To address this question, we followed the fate of i.v. injected IgM^+^IgD^+^ B cells, which are MHCII^intermediate^, CD80^−^ and Aire^−^. Seven days after injection, donor cells in the spleen had retained their initial phenotype. In contrast, cells that had immigrated into the thymus recapitulated all features of *steady state* thymic B cells, indicating that the unique phenotype of thymic B cells is imprinted by extrinsic cues in the thymic microenvironment, and we referred to this microenvironmental programing as “thymic B cell licensing” (9).

### Thymic B cell licensing requires CD40

The transition from a MHCII^int^CD80^−^Aire^−^stage to a MHCII^hi^CD80^+^Aire^+^ phenotype during thymic B cell licensing is strikingly reminiscent of mTEC “maturation.” However, whereas mTEC maturation is orchestrated by RANK signals (15–17), thymic B cell licensing crucially requires another TNF family member, CD40. Treatment of splenic B cells with agonist anti-CD40 antibody emulated B cell licensing, including induction of Aire (9). Thymic B cells in *Cd40*^−/−^ and *Cd40lg*^−/−^ mice are substantially diminished, consistent with a role of CD40 signals in thymic B cell homeostasis (7). Strikingly, the phenotype of thymic B cells in the absence of CD40 signaling is that of resting peripheral B cells. Thus, distinct signaling axes control the expression of Aire and the acquisition of a MHCII^high^CD80^+^ phenotype in mTECs or thymic B cells.

### Cross-talk with autoreactive CD4 T cells regulates thymic B cell numbers and licensing

**Figure 1:**
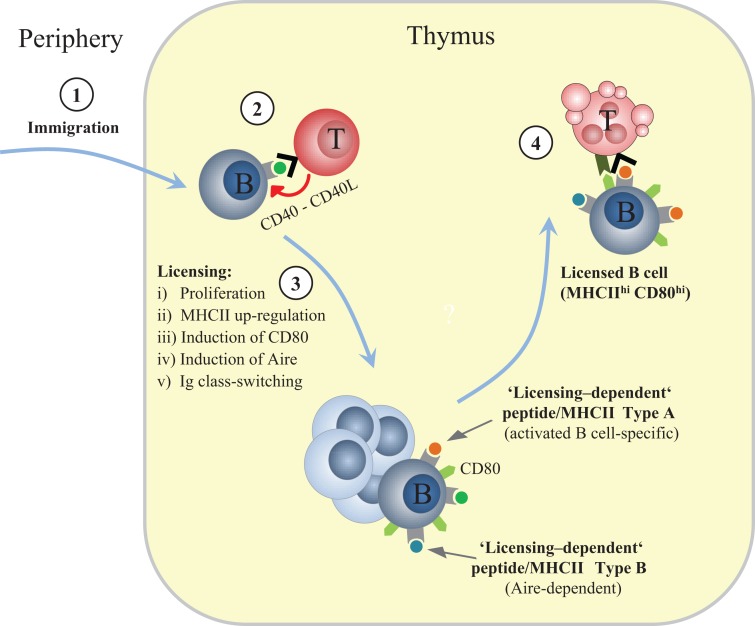
**Sequential phases of thymic B cell licensing**. (1) Recirculating peripheral B cells can enter the thymus. Their peptide (p)MHCII ligandome is expected to mostly contain endogenously expressed “resting B cell autoantigens.” Whereas the peripheral T cell repertoire is likely to be robustly tolerant toward these self-antigens, the nascent CD4 SP compartment is not (yet) fully purged of the respective specificities. (2) An unknown fraction of “B cell-reactive” CD4 SP cells within the diverse CD4 SP repertoire recognize “resting B cell autoantigens” and provide CD40 signals. CD40L is constitutively expressed by CD4 SP cells regardless of autoreactivity, presumably as a consequence of preceeding positively selecting interactions with cTECs. (3) Cross-talk with CD4 single-positive thymocytes induces proliferation, up-regulation of MHC class II, induction of CD80, induction of Aire, and Ig class-switching. (4) Licensed B cells delete autoreactive CD4 T cells, including TCR specificities that recognize “licensing-dependent” self-antigens; these are expected to include, but are not restricted to, self-antigens that are also up-regulated in activated B cells in the periphery (Type A) and Aire-dependent self-antigens (TRAs) (Type B).

Fujihara et al. showed that thymic B cell numbers are regulated by CD4 single-positive (SP) thymocytes (7). Similarly, we found that thymic B cells from TCRα^−/−^ mice, which lack SP thymocytes, express low levels of MHCII, are negative for co-stimulatory molecules, do not undergo class-switching and fail to up-regulate Aire (9). Strikingly, although CD4 SP thymocytes in “monoclonal” OT2 TCR transgenic mice on TCRα^−/−^ background express normal levels of CD40L, B cells in these mice are not licensed, indicating that a polyclonal repertoire of CD4 SP cells is necessary. The requirement for diverse TCRs seems to reflect a critical role of autoreactivity within the nascent CD4 SP compartment. Along these lines, B cell licensing can be mimicked *in vitro* when splenic B cells are pulsed with cognate antigen and co-cultured with specific CD4 SP cells. Moreover, MHCII-deficient B cells do not undergo licensing upon immigration into the thymus. Together, these observations suggest that cognate interactions through direct antigen presentation by B cells provide a platform for CD40 signaling and thereby initiate licensing (Figure 1). This tolerogenic feed-forward loop represents a striking parallel to the very similar cross-talk between mTECs and CD4 SP cells that is thought to bolster the tolerogenic features of mTECs (18).

## Thymic B Cells and Central T Cell Tolerance

### Evidence for a non-redundant contribution of thymic B cells to central tolerance

Several studies have shown that thymic B cells can contribute to negative selection under particular experimental conditions. Forced expression of the I-E MHCII molecule exclusively on B cells led to deletion of superantigen reactive T cells (19). Myelin oligodendrocyte glycoprotein (MOG)-specific CD4^+^ thymocytes were negatively selected when an epitope of MOG was exclusively presented by B cells (20). Perera et al. used autoreactive B cell receptor (BCR) transgenic mice to show that cognate T cells of the “same specificity” were negatively selected in the thymus (10). We showed that a “licensing-dependent” neo-antigen selectively up-regulated in immigrating B cells mediated negative selection through direct presentation (9).

What is known about the overall contribution of thymic B cells to central T cell tolerance? In mice lacking B cells, the size of the CD4SP cell compartment is significantly increased (9, 21). This resembles previous observations in mice that either lacked DCs or had a diminution of MHCII on mTECs, suggesting a non-redundant contribution of thymic B cells to negative selection of CD4 T cells (22, 23). Other recent reports showed that thymic Treg cells are decreased in B cell-deficient mice, and increased Treg numbers were observed in the thymus of Baff-transgenic mice harboring elevated B cell numbers (21, 24). Although these findings support a role of thymic B cell for central tolerance induction under physiological conditions, the exact spectrum of self-antigens that may require such a contribution remains to be characterized.

### Do thymic B cells pre-empt the self-antigen signature of germinal center B cells?

The role of CD40 and the cognate interactions between B cells and CD4 T cells during thymic B cell licensing are reminiscent of the germinal center (GC) reaction (25). Indeed, a substantial fraction of thymic B cells display a Fas^+^GL7^+^ phenotype that is otherwise characteristic for GC B cells (9). It is therefore tempting to speculate that the tolerogenic potential of licensed thymic B cells might in particular comprise “activated-B-cell” autoantigens. Consistent with that idea, CD4 SP thymoctes from B cell-deficient mice are hyper-responsive to CD40-activated B cells (9). A central assumption of the GC paradigm is that CD4 T cell help needs to be tightly focused on epitopes of the foreign antigen that has been internalized via the BCR (26). In order to control BCR–hypermutation-related neo-autoreactivity among GC B cells, self-reactive B cells need to be deprived from cognate help. This not only requires robust CD4 T cell tolerance toward exogenously derived self-determinants that have been captured via the hypermutated BCR but also that the CD4 T cell repertoire is efficiently purged of reactivity toward any endogenously derived B cell autoantigen that is concurrently presented. Because thymic B cells may emulate the peptide/MHC composition of GC B cells in a tolerogenic setting, thymic B cell licensing may pre-empt T cell recognition of “activated B cell autoantigens” in an inflammatory context in secondary lymphoid tissues.

### A role for the BCR?

B cells efficiently present antigens that have been captured via the BCR (27). So, do BCR-specificity and/or -autoreactivity play a role in thymic B cell licensing and B cell-mediated central tolerance? Using BCR knock-in and T cell receptor transgenic mice specific for the same antigen, Perera et al. elegantly demonstrated that autoreactive B cells are particularly efficient APCs to induce negative selection of T cells with identical specificity (10). However, we found no evidence that BCR autoreactivity may favor B cell entry into the thymus or is a prerequisite to subsequently undergo licensing (9). Specifically, we employed SW_HEL_ mice to address these issues. In these mice, around two-thirds of peripheral B cells express a transgenic “anti-foreign BCR” specific for hen egg lysozyme (HEL), whereas the remainder of B cells carry endogenously rearranged BCRs, some of which may harbor autoreactivity. The relative abundance of HEL^+^ B cells in the thymus exactly reflected their peripheral frequency, and both HEL^+^ and HEL^−^ B cells underwent intrathymic licensing. Future work is needed to more conclusively address the composition of the thymic B cell repertoire, for instance, through BCR sequencing.

## Open Questions

### Is BCR class-switching in the thymus physiologically relevant?

As a consequence of licensing, thymic B cells class-switch to IgG or IgA. It is therefore conceivable that thymic B cells present B lineage-specific “neo-epitopes” generated through isotype-class-switching or possibly also somatic hypermutation. In all likelihood, and in distinction from the GC reaction, class-switching in the thymus may occur “spontaneously,” i.e., independent of cognate help downstream of BCR-mediated antigen capture. This has obvious implications for the emergence of “natural” Igs. Some thymic B cells display a memory B cell phenotype (CD38^+^Fas^lo^), and it is possible that IgG- or IgA-positive thymic B cells re-enter the blood stream. In fact, we observed a minute population of class-switched donor cell in the periphery after intrathymic injection of naive B cells (Tomoyoshi Yamano and Ludger Klein unpublished). More work is needed to clarify the role of the thymus as a potential source of natural Igs.

### Do thymic B cells shape the thymic microenvironment?

It was shown that thymic B cells express Ltα and Ltβ, and thereby regulate mTEC cellularity (28). Thymic B cells also express a variety of cytokines and chemokines, e.g., IL-10, IL-12, IL-16, and CCL22 (Tomoyoshi Yamano and Ludger Klein unpublished). These data suggest additional layers of cross-talk in the thymus. However, more work is needed to better understand whether and how thymic B cells may organize the thymic microenvironment.

### Do thymic B cells play a role in human disease?

The presence of B cells with an “activated” phenotype in the healthy human thymus has long been recognized (29), but their origin and specificity has remained elusive. Ectopic GC-like structures are present in thymi of early-onset myasthenia gravis (MG) patients, and it was hypothesized that they are the site of auto-sensitization against the acetylcholine receptor (AChR) (30). However, a recent characterization of antibody repertoires in MG thymi revealed expansion of a polyclonal repertoire unrelated to AChR specificities (31). Thus, it remains to be shown whether the accumulation of B cells in MG thymi is a cause or a consequence of disease.

## Conflict of Interest Statement

The authors declare that the research was conducted in the absence of any commercial or financial relationships that could be construed as a potential conflict of interest.
